# AR/PCC herb pair inhibits osteoblast pyroptosis to alleviate diabetes‐related osteoporosis by activating Nrf2/Keap1 pathway

**DOI:** 10.1111/jcmm.17928

**Published:** 2023-08-24

**Authors:** Fangda Fu, Huan Luo, Yu Du, Yuying Chen, Kun Tian, Jin Pan, Jian Li, Nani Wang, Ronghua Bao, Hongting Jin, Peijian Tong, Hongfeng Ruan, Chengliang Wu

**Affiliations:** ^1^ Institute of Orthopaedics and Traumatology The First Affiliated Hospital of Zhejiang Chinese Medical University (Zhejiang Provincial Hospital of Traditional Chinese Medicine) Hangzhou China; ^2^ Department of Pharmacy, The Second Affiliated Hospital, School of Medicine Zhejiang University Hangzhou China; ^3^ The First Affiliated Hospital of Zhejiang Chinese Medical University Hangzhou China; ^4^ The Fourth Clinical Medical College of Zhejiang Chinese Medical University Hangzhou China; ^5^ Department of Orthopaedics The First Affiliated Hospital of Zhejiang Chinese Medical University Hangzhou China; ^6^ Department of Architecture, School of Architecture China Academy of Art Hangzhou China; ^7^ Department of Orthopaedics Hangzhou Ninth People's Hospital Hangzhou China; ^8^ Department of Medicine Zhejiang Academy of Traditional Chinese Medicine Hangzhou China; ^9^ Hangzhou Fuyang Hospital of TCM Orthopedics and Traumatology Hangzhou China

**Keywords:** Anemarrhenae Rhizoma/Phellodendri Chinensis Cortex herb pair, diabetes, Nrf2/Keap1 signalling, osteoporosis, pyroptosis

## Abstract

Osteoporosis is a prevalent complication of diabetes, characterized by systemic metabolic impairment of bone mass and microarchitecture, particularly in the spine. Anemarrhenae Rhizoma/Phellodendri Chinensis Cortex (AR/PCC) herb pair has been extensively employed in Traditional Chinese Medicine to manage diabetes; however, its potential to ameliorate diabetic osteoporosis (DOP) has remained obscure. Herein, we explored the protective efficacy of AR/PCC herb pair against DOP using a streptozotocin (STZ)‐induced rat diabetic model. Our data showed that AR/PCC could effectively reduce the elevated fasting blood glucose and reverse the osteoporotic phenotype of diabetic rats, resulting in significant improvements in vertebral trabecular area percentage, trabecular thickness and trabecular number, while reducing trabecular separation. Specifically, AR/PCC herb pair improved impaired osteogenesis, nerve ingrowth and angiogenesis. More importantly, it could mitigate the aberrant activation of osteoblast pyroptosis in the vertebral bodies of diabetic rats by reducing increased expressions of Nlrp3, Asc, Caspase1, Gsdmd and IL‐1β. Mechanistically, AR/PCC activated antioxidant pathway through the upregulation of the antioxidant response protein Nrf2, while concurrently decreasing its negative feedback regulator Keap1. Collectively, our in vivo findings demonstrate that AR/PCC can inhibit osteoblast pyroptosis and alleviate STZ‐induced rat DOP, suggesting its potential as a therapeutic agent for mitigating DOP.

## 1 INTRODUCTION

Diabetes mellitus (DM) is a chronic metabolic disease characterized by abnormal insulin‐dependent glucose metabolism,^1^ which disrupts the homeostasis of bone minerals, such as calcium, phosphorus and magnesium, leading to diminished bone mass and structural deterioration.^2^ Accumulating clinical evidence demonstrates a threefold increase in fracture incidence among approximately 50%–65% of DM patients, with nearly 35% diagnosed as diabetic osteoporosis (DOP).^3^, ^4^ Despite progress in DOP management in recent decades, concerns regarding the long‐term side effects (such as atypical fracture, muscle and joint pain, and increased risk of stroke) of antiresorptive drugs such as bisphosphonates, denosumab and odanacatib, coupled with insufficient evidence on their sustained efficacy, have resulted in inadequate patient adherence.^5^ Therefore, there is a pressing clinical need to continue developing drugs that offer prolonged anti‐DOP effects while minimizing side effects.

Anemarrhenae Rhizoma (AR, derived from *Anemarrhena asphodeloides* Bunge) and Phellodendron Chinensis Cortex (PCC, obtained from the bark of *Phellodendron chinense* C. K. Schneid.) were initially documented in the Secret Record of the Chamber of Orchids.^6^ This herb pair, composed of AR and PCC in a specific ratio, is distinguished by its ability to tonify ‘kidney’ and nourish ‘Yin’, and has long been implemented in Traditional Chinese Medicine for DM treatment, such as in Zishen pill.^7^ Growing evidence indicates that this herb pair exhibits a remarkable therapeutic effect against DOP.^8^, ^9^, ^10^ In line with this, a previous study conducted by us demonstrated the hypoglycaemic and anti‐osteoporotic properties of the AR/PCC herb pair in a diabetic zebrafish model, primarily regulating osteoblast activities.^11^ However, the precise mechanism by which the AR/PCC herb pair modulates osteogenic differentiation and bone formation in the context of DOP remains obscure.

The DOP progression is widely acknowledged to involve multifactorial factors, including increased osteoclast differentiation, compromised nutritional support and heightened oxidative stress.^12^ Accruing works substantiate that hyperglycaemia amplifies pro‐inflammatory cytokines levels while diminishing the expression of osteogenic proteins crucial for bone quality. These proteins include runt‐related transcription factor 2 (Runx2) and Osterix, osteoblast‐secreted proteins that regulate bone resorption or bone remodelling such as Osteoprotegerin (Opg) and Osteocalcin (Oc), and Collagen 1 (Col1), which constitute 90% of the total organic component of bone matrix.^13^, ^14^, ^15^, ^16^ Furthermore, an increasing body of evidence highlights the involvement of the nervous and angiogenic systems in bone homeostasis, governing innervation, neovascularization and the distribution of oxygen, nutrients, immune cells and waste removal in bone tissue.^17^, ^18^, ^19^ A recent study utilizing streptozotocin (STZ)‐induced DOP mice demonstrated that a correlation between the loss of calcitonin gene‐related peptide positive (Cgrp^+^) sensory nerves and STZ‐induced bone loss,^17^ and further analysis revealed the role of Netrin‐1, a chemical attractant in axon guidance, in guiding the elongation of Cgrp^+^ sensory neurons and regulating bone remodelling processes.^18^ In parallel, reduced expression of platelet endothelial cell adhesion molecule (Cd31) and vascular endothelial growth factor (Vegf), indicative of diminished blood vessel density, was observed in the bone tissues of diabetic mice, contributing to decreased expression of osteogenic genes (*Runx2* and *Oc*).^20^ Beyond that, hyperglycaemia‐mediated lipid accumulation, stemming from decreased leptin and leptin receptor levels leads to the generation of glycation end products within the bone and affects the balance of osteoclasts and osteoblasts activity.^2^, ^21^

Pyroptosis represents a novel form of programmed cell death, primarily triggered by NLR Family Pyrin Domain Containing 3 (Nlrp3) inflammasome. It is characterized by activation of Caspase1 and Gasdermin D (Gsdmd)‐mediated membrane rupture, resulting in the excessive release of pro‐inflammatory mediators, particularly interleukin‐1β (IL‐1β).^22^ Emerging evidence shows that the chronic inflammatory microenvironment induced by hyperglycaemia activates the NLRP3 inflammasome and promotes the production of inflammatory factors, leading to the inhibition of proliferation and differentiation of osteoblasts; however, the silencing of Nlrp3 or inhibition of Caspase1 in osteoblasts could restore their ability to proliferate, differentiate and protect against bone mineralization deficiency in DM mice.^23^, ^24^, ^25^ Of note, AR and PCC have been documented to ameliorate inflammatory responses in a variety of diabetic complications, such as diabetic nephropathy and diabetes‐induced cognitive impairment, through the reduction in Nlrp3 and Caspase1 levels.^26^, ^27^ Based on the above findings, it is reasonable to speculate that AR/PPC herb pair may yield a similar role in attenuating osteoblast pyroptosis and mitigating the development of DOP.

As a pivotal modulator of oxidant–antioxidant equilibrium, the nuclear factor erythroid 2‐related factor 2 (Nrf2) plays a crucial role in upholding cellular redox homeostasis by impeding the binding of Kelch‐Like ECH‐Associated Protein 1 (Keap1) and governing the expression of antioxidant genes.^28^ Overwhelming evidence confirms that exposure to high glucose inhibits Nrf2/Keap1 signalling, leading to oxidative stress induction, assembly of Nlrp3, activation of Caspase1, and subsequent initiation of pyroptosis. This cascade culminates in disrupted bone remodelling and exacerbated osteoporosis; conversely, activation of Nrf2/Keap1 signalling preserves cellular redox homeostasis, impeding the cytotoxicity effects induced by high glucose in osteoblasts.^29^, ^30^, ^31^, ^32^ In addition, pharmacological investigations conducted in high fat‐induced obese rats have demonstrated that the active components of AR/PPC possess the capability to curb inflammation response by upregulating Nrf2 protein levels.^33^ Taken together, these studies support the notion that the administration of AR/PCC exhibits the potential to attenuate osteoblast pyroptosis and curb the progression of DOP through the activation of the Nrf2/Keap1 signalling pathway.

In this study, to investigate the therapeutic effect of AR/PCC herb pair in the progression of DOP, we constructed an STZ‐induced DOP model in rats and found that STZ‐treated DM rats exhibited elevated fasting blood glucose (FBG) levels along with a striking DOP phenotype, and administration of AR/PCC herb pair effectively reversed these manifestations. Subsequent analyses showed that AR/PCC exerts inhibitory effects on Nlrp3‐mediated pyroptosis in osteoblasts by activating the Nrf2/Keap1 signalling pathway. Our in vivo findings provide further insights into the pharmacological properties of AR/PCC concerning the progression of DOP, highlighting the potential of AR/PCC as a promising therapeutic agent for DOP treatment.

## 2 MATERIALS AND METHODS

### 2.1 Chemicals and reagents

Crude drugs of AR and PCC were provided by Zhejiang Jingyuetang Pharmaceutical Co. Ltd. Voucher specimens of AR (no. RA2019090821) and PCC (no. PA2019052101) were deposited at the Department of Medicine, Zhejiang Academy of Traditional Chinese Medicine. The primary antibodies used in this study are summarized in Table 1. The fluorescent‐conjugated secondary antibody was obtained from Sungene Biotech Co. The Alp stain kit (#D001‐2‐2) was purchased from Nanjing Jiancheng Bioengineering Institute (Nanjing, Jiangsu, China). Unless otherwise specified, all chemicals were acquired from Sigma‐Aldrich.

**TABLE 1:** Details information of primary antibodies used in this study.

Antibody	Host species	Dilution	Catalogue #	Company
Runx2	Rabbit	1:500	RLT4192	Ruiying Biological
Osterix	Rabbit	1:500	ab209484	Abcam
Opg	Rabbit	1:500	RLT3466	Ruiying Biological
Oc	Rabbit	1:500	ab13418	Abcam
Col1	Rabbit	1:500	RLT1018	Ruiying Biological
Netrin	Rabbit	1:500	ab39370	Abcam
Cgrp	Rabbit	1:500	ab81887	Abcam
Vegf	Rabbit	1:500	RLT4870	Ruiying Biological
Cd31	Rabbit	1:500	RLT0752	Ruiying Biological
Nlrp3	Rabbit	1:500	19771‐1‐AP	Proteintech
Caspase1	Rabbit	1:500	22915‐1‐AP	Proteintech
Gsdmd	Rabbit	1:500	ab219800	Abcam
IL‐1β	Rabbit	1:500	bs6319R	Bioss
Nrf2	Rabbit	1:500	YT3189	Immunoway
Keap1	Rabbit	1:500	YT5218	Immunoway
Lep	Rabbit	1:500	bs20498R	Bioss
Lepr	Rabbit	1:500	bs0409R	Bioss

### 2.2 Preparation of herbal extracts

AR/PCC pair was prepared in a 1:1 (w/w) ratio, as we previously described.^34^ Briefly, AR and PCC were blended and subjected to two rounds of extraction using 10‐fold water (w/v) at 100°C for 2 h each time. The filtrates were subsequently lyophilized using a SCIENTZ‐12N/A freezing dryer system (Ningbo Scientz Co. Ltd.) and dissolved in citrate buffer (pH = 4.4) prior to oral gavage.

### 2.3 Animal treatment

Eight‐week‐old male Wistar rats, weighing 200–220 g, were procured from Beijing Vita River Laboratory Animal Technology (Beijing, China). All rats were housed in a temperature‐controlled room at 23 ± 2°C within a 12‐h light/dark cycle, and they had ad libitum access to water and lab chow. All experiments were approved by The Ethics Committee of Zhejiang Traditional Chinese Medicine Institution and performed in accordance with the ethical guidelines for using and caring of laboratory animals with the permission of Zhejiang Traditional Chinese Medicine Institution principles for laboratory animal use and care (no. [2019]018).

All rats were randomly divided into four groups (*n* = 6 per group): Control group, Model group, Low AR/PCC group and High AR/PCC group. All rats except those in the Control group underwent DOP modelling. The STZ‐induced DOP model was established as we previously described.^11^, ^34^, ^35^ Briefly, rats received a single intraperitoneal injection of STZ (35 mg/kg). The concentration of FBG in rats was monitored using a blood glucometer (Yuyue Instrument Co. Ltd). Rats with FBG >16.7 mmol/L were considered diabetic and randomly assigned to the Model group, Low AR/PCC group and High AR/PCC group. The rats in the Low AR/PCC group and High AR/PCC group received oral gavage of 4 or 8 g/kg AR/PCC extract per day, respectively, while the rats in the Control group and Model group received an equivalent volume of citrate buffer. After 12 weeks of treatment, all rats were sacrificed following an overnight fast, and serum samples and lumbar vertebrae were harvested for further evaluation. Mass spectrometry verification of the seven absorbable components of the AR/PCC herbal pair in rat serum is available in our previous study.^34^

### 2.4 Pathological staining

All rat lumbar vertebrae were fixed in 4% paraformaldehyde for 48 h. After rinsing with PBS, the vertebrae were decalcified in 14% EDTA solution for 8 weeks and then cut into 5‐μm‐thick sections. The sections were stained with haematoxylin and eosin solutions and captured under a microscope (Carl Zeiss). Bone morphometric parameters including trabecular area (Tb.Ar), trabecular number (Tb.N), trabecular thickness (Tb.Th) and trabecular separation (Tb.Sp) were analysed using the Image‐Pro Plus Software version 6.0 (Media Cybernetics Inc). The histological scoring was conducted by a blind pathologist, following a previously described method.^36^

### 2.5 Histological Alp assay

The sections were deparaffinized in xylene, rehydrated by a graded series of alcohol, rinsed with PBS and then stained with an Alp kit as per the manufacturer's instructions. Triplicates of each sample were used for staining. The Alp‐positive staining area in the 4th lumbar vertebral body was analysed and quantified using a microscope (Carl Zeiss).

### 2.6 Immunofluorescence analysis

Sections were deparaffinized, rehydrated and subjected to antigen retrieval in 0.01 mol/L citrate buffer at 60°C for 6 h. Then, sections were treated with 0.3% hydrogen peroxide to reduce endogenous peroxidase activity and blocked with 5% normal goat serum for 30 min at room temperature. The sections were then incubated with respective primary antibody overnight at 4°C, and negative control sections were incubated with PBS. Subsequently, they were incubated with fluorescent‐conjugated secondary antibody for 1 h in the dark. Nonspecific fluorescence in the vertebral body was quenched with Vector® TrueVIEW® Autofluorescence Quenching Kit (Vector Laboratories Inc). After DAPI counterstaining, the sections were observed under the fluorescence microscope (Carl Zeiss). Each experiment was repeated in triplicates. Quantitative histomorphometric analysis was performed using the Image‐Pro Plus Software version 6.0, and the integrated optical density in indicated areas was calculated in a blinded manner. The percentages of Cgrp^+^ cells and Cd31^+^ cells were determined by counting the positively stained region in the indicated areas as we previously described.^37^

### 2.7 Statistical analysis

All numerical data were expressed as mean ± SEM. One‐way analysis of variance (anova) followed by Tukey's multiple comparisons test was performed using the GraphPad Prism 8 statistics software, to compare the statistical differences among groups. *p* < 0.05 was considered as statistical significance.

## 3 RESULTS

### 3.1 AR/PCC herb pair reduces FBG levels and inhibits lipid synthesis in the vertebral bodies of DM rats

**FIGURE 1:**
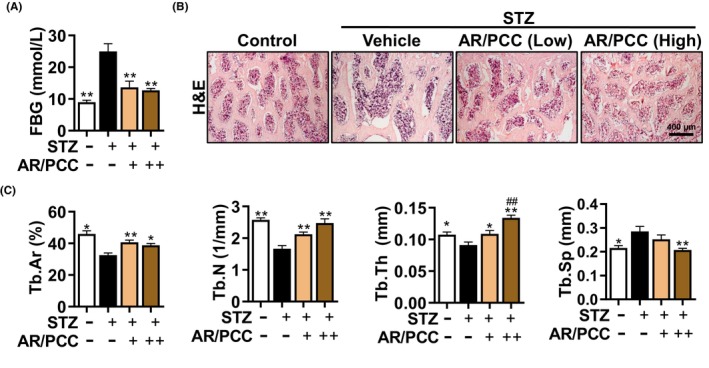
AR/PCC herb pair reduces blood glucose levels and improves the trabecular bone environment in DM rats. (A) The FBG levels were assessed after 12 weeks of intraperitoneal injection of 35 mg/kg STZ. (B) Haematoxylin and eosin staining of the vertebral body. (C) Calculation of bone morphometric parameters in vertebral body in (B). Data are presented as mean  ± SEM. Each experiment was performed in triplicate. *n* = 6 per group. *p < 0.05, **p < 0.01, compared to the Model group; ^##^ *p* < 0.01, compared to the low‐dose AR/PCC group.

It is well‐established that STZ could impair pancreatic islet beta‐cell, serving as a widely used method to construct rat DM models.^11^, ^34^, ^35^ To investigate the pharmacological effect of AR/PCC on DOP progression, STZ‐induced DM rats (FBG > 16.7 mmol/L) were established and administered with AR/PCC extract orally on a daily basis for 12 weeks. We found that significantly higher FBG levels in DM rats compared to the Control rats (24.98 vs. 8.98 mmol/L), and treatment with AR/PCC significantly reduced FBG levels to comparable levels in the Control rats (Figure 1A).

It has been documented that diabetic patients generally have normal weight in the early stages, but as the DM progresses, more than three‐quarters of patients exhibit abnormal lipid metabolism, which can be regulated by impaired expression of Lep and Lepr.^38^ Herein, we found no significant changes in the body weight of rats among the different groups (Figure S1A). To evaluate the impact of AR/PCC on the lipid metabolism of DM rats, we analysed the expression of Lep and Lepr, two indicators of lipid synthesis, using immunofluorescence (IF) analysis. The results showed that AR/PCC could significantly overturn the decreased expression of Lep and Lepr proteins in the vertebral bodies of DM rats (Figure S1B). These results indicate that AR/PCC herb pair can effectively reduce blood glucose levels and improve lipid synthesis in the vertebral bodies of DM rats.

### 3.2 AR/PCC improves trabecular bone architecture and accelerates osteogenesis in the vertebral bodies of DM rats

The main manifestation of DOP is impaired trabecular bone structure associated with hyperglycaemia, which increases the risk of bone fracture.^2^ To assess whether AR/PCC can improve the trabecular bone quality of DM rats, the morphological changes of the 4th lumbar vertebral bodies were detected using haematoxylin and eosin staining. We found that AR/PCC significantly mitigated bone loss in DM rats (Figure 1B). In parallel, analysis of bone morphological parameters revealed that AR/PCC dose‐dependently reversed the reduction in Tb.Th and Tb.N, as well as the elevation of Tb.Sp, while only partially restoring the reduced Tb.Ar (Figure 1C).

**FIGURE 2:**
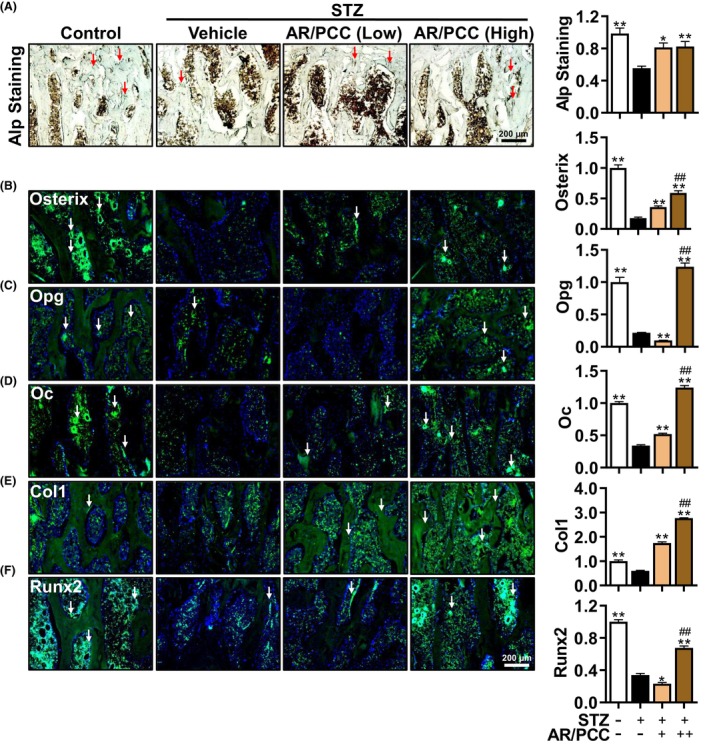
AR/PCC herb pair accelerates osteogenesis in DM rats. (A) The Alp staining of the vertebral body. Red arrows indicate the area of Alp‐positive bone formation. (B–F) IF staining and quantitative expression analysis of osteogenesis‐related proteins (Osterix, Runx2, Opg, Oc, Col1) in the vertebral body. DAPI stains nuclei blue. White arrows indicate strong positive expression in the bone formation area. Data are presented as mean  ± SEM. Each experiment was performed in triplicate. *n* = 6 per group. **p < 0.01, compared to the Model group; ^##^ *p* < 0.01, compared to the low‐dose AR/PCC group.

Emerging evidence from clinical studies suggests that diabetes could lead to decreased proliferation and differentiation potential in osteoblasts.^39^ To identify whether AR/PCC can improve impaired osteogenesis in DM rats, the Alp activity in the vertebral body was determined using an Alp activity kit. Consistent with haematoxylin and eosin staining results, we found a significant reduction in the Alp activity in the lumbar vertebral body of the Model group (down to 56.3% of the Control group), and low‐dose and high‐dose AR/PCC treatment could reverse the decrease in Alp activity to 82.6% and 83.5% of the Control group, respectively (Figure 2A). To confirm these findings, we evaluated the expression of osteogenic‐related proteins (Osterix, Runx2, Opg, Oc, Col1) using IF analysis. As expected, we found that AR/PCC could dose‐dependently increase the expressions of Osterix, Opg, Oc, and Col1 in the lumbar vertebral bodies of DM rats (Figures 2B–E). Intriguingly, although high‐dose AR/PCC administration significantly improved the reduction of Runx2 in DM rats, the low‐dose AR/PCC further disrupted its expression (Figure 2F). Our findings indicate that AR/PCC can ameliorate impaired osteogenesis in DM rats.

### 3.3 AR/PCC promotes nerve ingrowth and angiogenesis in the vertebral bodies of DM rats

**FIGURE 3:**
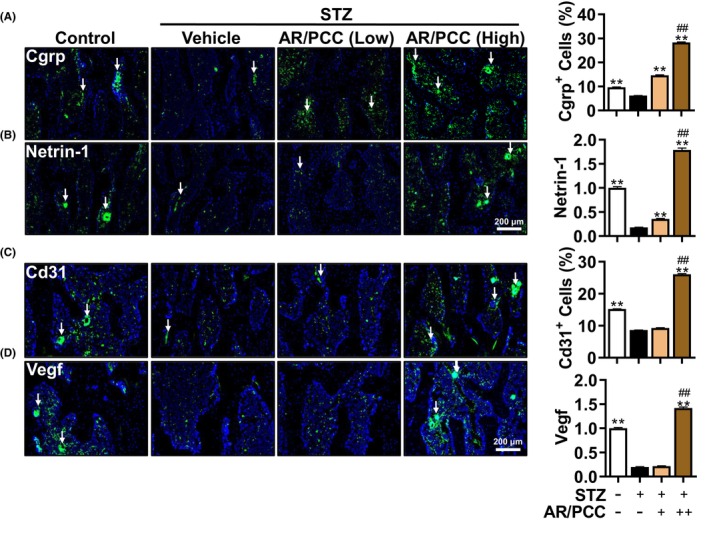
AR/PCC herb pair promotes nerve ingrowth and angiogenesis in the vertebral body of DM rats. (A, B) IF staining and quantification of Netrin1 and Cgrp expression in the marrow cavity. DAPI stains nuclei blue. White arrows indicate Netrin1‐ or Cgrp‐positive cells in the marrow cavity. (C, D) IF staining and quantification expression of Vegf and Cd31 in the marrow cavity. DAPI stains nuclei blue. White arrows indicate Vegf‐ or Cd31‐positive cells in the marrow cavity. Data are presented as mean  ± SEM. Each experiment was performed in triplicate. *n* = 6 per group. **p < 0.01, compared to the Model group; ^##^ *p* < 0.01, compared to the low‐dose AR/PCC group.

It has been well‐established that osteogenesis is closely coupled with nerve ingrowth during bone remodelling, and the loss of Cgrp^+^ sensory neurons is correlated with decreased osteogenic potential in DOP mice.^17^, ^18^ To further investigated the potential effects of AP/RCC on sensory nerve ingrowth in the vertebral bodies of DM rats, we examined the number of Cgrp^+^ cells in the vertebral body, and expression changes of Netrin‐1, an axonal guidance molecule secreted by osteoclasts, using IF analysis. Compared with the Control rats, DM modelling significantly reduced the number of Cgrp^+^ sensory neurons and Netrin‐1 levels in the vertebral bodies of DM rats, which were dose‐dependently reversed by AR/PCC. Interestingly, high‐dose AR/PCC treatment resulted in a higher density of Cgrp^+^ sensory neurons and Netrin‐1 levels compared with the Control group (Figure 3A,B).

Besides, it has been documented that hyperglycaemia‐induced accumulation of glycation end products could lead to a reduction of blood vessels within the marrow cavity, consequently downregulating osteogenic genes (*Runx2* and *Oc*) in DM mice.^19^, ^20^ Then, we analysed whether AR/PCC affects angiogenesis in the vertebral body of DM rats. The IF results of Cd31 and Vegf, which serve as markers for angiogenesis, showed that AR/PCC could effectively mitigate the reduced number of Cd31^+^ cells and the decline of Vegf levels within the vertebral bodies of DM rats in a dose‐dependent manner (Figure 3C,D). Unexpectedly, high AR/PCC treatment resulted in higher numbers of Cd31^+^ cells as well as increased Vegf expression compared to the Control group. Taken together, these results substantiate that AR/PCC extracts could improve innervation and angiogenesis within the vertebral bodies of DM rats, thereby resisting the progression of osteoporosis.

### 3.4 AR/PCC inhibits Nlrp3‐mediated pyroptosis of osteoblasts within the vertebral bodies of DM rats

**FIGURE 4:**
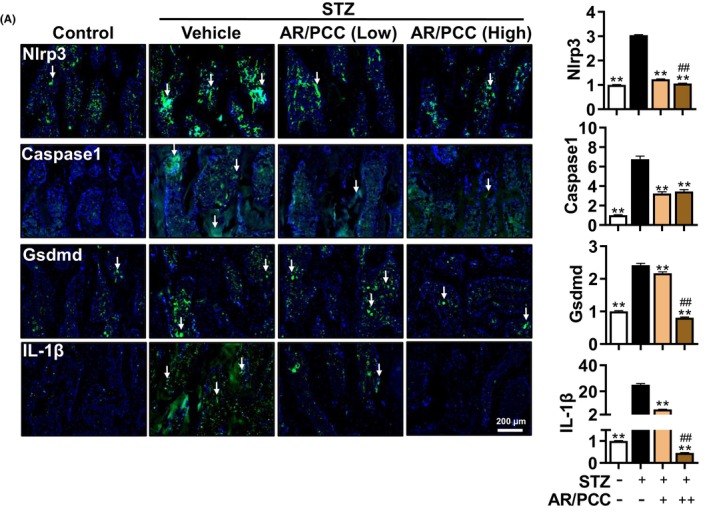
AR/PCC herb pair inhibits osteoblast pyroptosis in the vertebral body of diabetic rats. IF staining and quantification of Nlrp3, Caspase1, Gsdmd, and IL‐1β expression in bone formation area. DAPI stains nuclei blue. White arrows indicate positive expression of Netrin1 or Cgrp in the bone formation area. Data are presented as mean  ± SEM. Each experiment was performed in triplicate. *n* = 6 per group. **p < 0.01, compared to the Model group; ^##^ *p* < 0.01, compared to the low‐dose AR/PCC group.

Emerging evidence suggests that the NLRP3 inflammasome plays a vital role in bone formation and the pathogenesis of osteoporosis by affecting the differentiation of osteoblasts and osteoclasts through autocrine and paracrine manners.^40^ Moreover, hyperglycaemia can promote chronic low‐grade inflammation mediated by the Nlrp3 inflammasome, contributing to the pathogenesis of DM and its complications.^41^ To identify the effect of AR/PCC on osteoblast pyroptosis, we analysed the expressions of key pyroptosis‐related proteins, Nlrp3, Caspase1, Gsdmd and IL‐1β, within the vertebral body using IF analyses. We found a significant increase in the expression levels of Nlrp3, Caspase1, Gsdmd and IL‐1β within the vertebral bodies of DM rats, and AR/PCC extracts could dose‐dependently overturn these alterations (Figure 4). It is noteworthy that high‐dose AR/PCC treatment reduced the elevated levels of Nlrp3, Gsdmd, and IL‐1β to levels comparable to those of the Control group. The above findings suggest that AR/PCC administration may alleviate the progression of osteoporosis in DM rats primarily by inhibiting osteoblast pyroptosis‐mediated inflammation.

### 3.5 AR/PCC activates Nrf2/Keap1 signalling pathway in the vertebral body of DM rats

**FIGURE 5:**
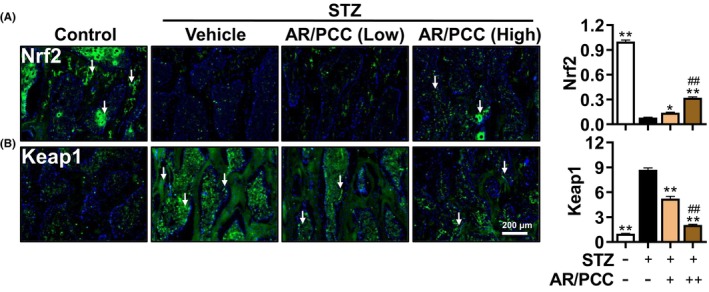
AR/PCC herb pair activates the Nrf2/Keap1 pathway in the vertebral body of DM rats. (A, B) IF staining and quantification of Nrf2 and Keap1 expression in bone formation area. DAPI stains nuclei blue. White arrows indicate positive expression of Netrin1 or Cgrp in the bone formation area. Data are presented as mean  ± SEM. Each experiment was performed in triplicate. *n* = 6 per group. *p < 0.05, **p < 0.01, compared to the Model group; ^##^ *p* < 0.01, compared to the Low‐dose AR/PCC group.

An increasing body of evidence suggests that cellular oxidant damage is a significant trigger for the activation of Nlrp3‐mediated pyroptosis, which is mediated by the Nrf2/Keap1 signalling.^30^, ^42^ As a main regulator of the antioxidant response system, the Nrf2/Keap1 signalling plays a crucial role in maintaining cellular homeostasis under stressful or inflammatory conditions in various cell types, including osteoblasts and osteoclasts.^43^, ^44^ To better understand the underlying mechanism by which AR/PCC improves osteoblast pyroptosis in DM rats, the expressions of Nrf2 and Keap1 (the negative feedback regulator of Nrf2) were determined by IF analysis. The IF results showed a 92% reduction in Nrf2 expression and an 8.7‐fold increase in Keap1 expression in DM rats compared with the Control rats, while AR/PCC could significantly reverse these alterations in a dose‐dependent manner in DM rats (Figure 5A,B). Noteworthy, the Keap1 in AR/PCC high group was reduced to a level comparable to that in the Control group, but Nrf2 only increased to 32% of the Control rats (Figure 5A,B), indicating that Keap1 might be the primary target for AR/PCC. Overall, these findings suggest that AR/PCC herb pair can inhibit osteoblast pyroptosis and ameliorate the progression of osteoporosis in the vertebral bodies of DM rats by activating the Nrf2/Keap1 signalling pathway.

## 4 DISCUSSION

**FIGURE 6:**
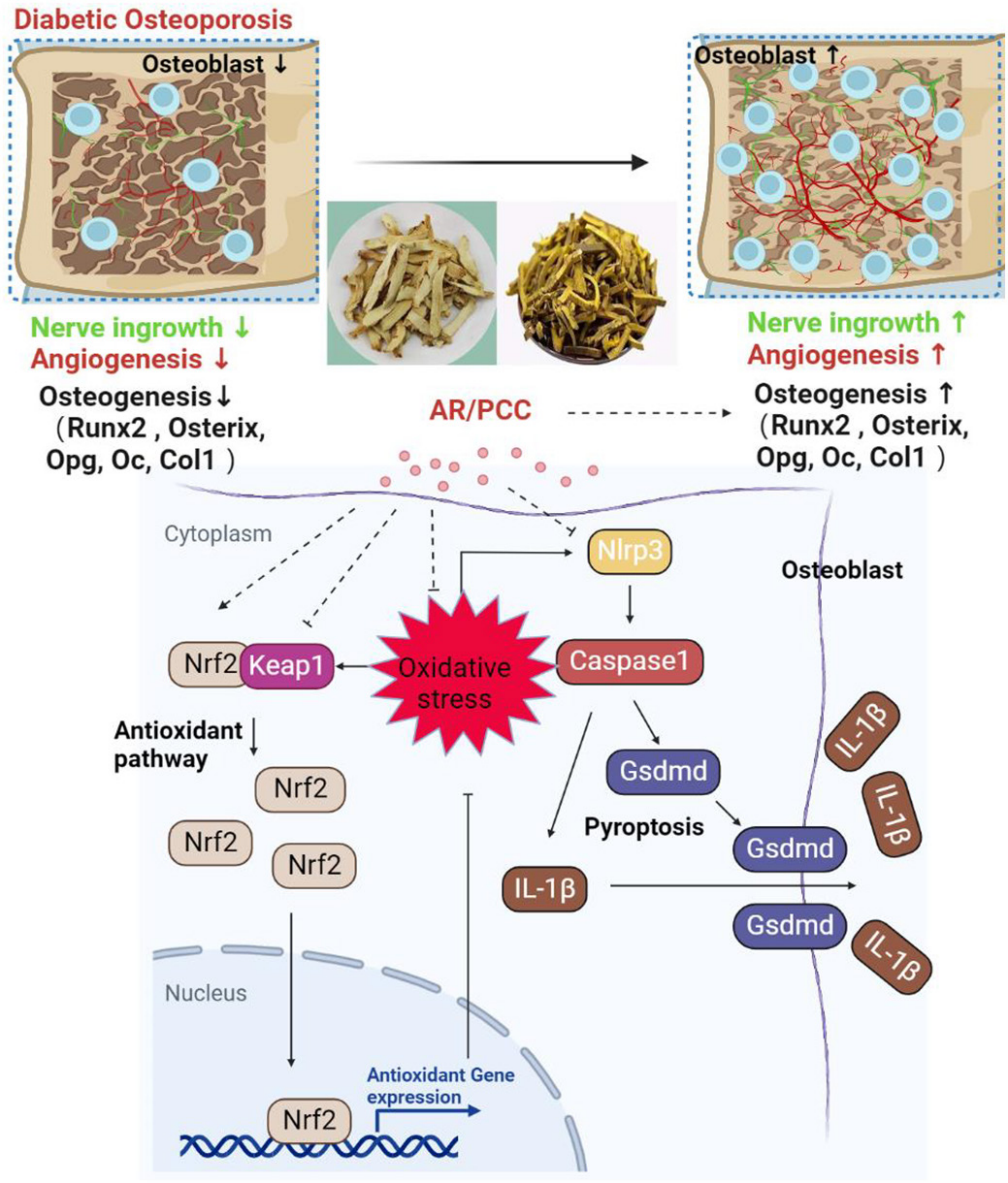
Schematic representation of the working model illustrating how the AR/PCC herb pair ameliorates STZ‐induced DOP by inhibiting osteoblast pyroptosis via activation of the Nrf2/Keap1 signalling pathway.

Diabetic osteoporosis is a progressive diabetic complication, and the available therapies for DOP are limited in number and efficacy.^5^ The AR/PCC is a traditional Chinese herbal pair that has been widely utilized for the management of DM.^9^, ^27^, ^45^ Importantly, both our latest discoveries and other studies using zebrafish or rodent DM models have demonstrated the effective attenuation of osteoporotic phenotypes by AR/PCC or certain AR/PCC‐derived compounds.^8^, ^9^, ^10^, ^11^ Based on these findings, we hypothesized that AR/PCC exhibits beneficial properties against DOP progression. In this study, we utilized the STZ‐induced DM model in rats to assess the pharmacological effect of AR/PCC on the DOP phenotype. Our results showed that AR/PCC administration not only reduced the increased FSG levels in DM rats but also ameliorated osteoporotic phenotype within the vertebral bodies by improving bone loss and osteogenic capacity while promoting nerve ingrowth and angiogenesis. Subsequent mechanistic analysis revealed that AR/PCC could suppress the Nlrp3‐mediated pyroptosis of osteoblasts through activation of the Nrf2/Keap1 signalling pathway. To the best of our knowledge, our findings provide the first in vivo evidence of the pharmacological effects of AR/PCC herb pair on DOP development by targeting osteoblast pyroptosis, corroborating that AR/PCC is a promising therapeutic agent for the treatment of DOP (Figure 6).

Although the pathogenesis of DOP has not been fully elucidated, a persistent hyperglycaemic environment can stimulate Nlrp3 expression and inflammasome assembly, which is one of the most important contributors to the pathogenesis of DM and its associated complications, including diabetic nephropathy, diabetic retinopathy and diabetic cardiomyopathy.^46^, ^47^, ^48^ Accumulating evidence from in vivo and in vitro evidence suggests that the elevated glucose induces low‐grade systemic inflammation and pyroptosis in osteoblasts, while the overexpression of Nlrp3 protein aggravates bone resorption and impairs osteogenesis, and targeting Nlrp3 or its associated components, such as Caspase‐1, IL‐1β and IL‐18, can effectively reverse the inhibition of osteoblast proliferation and differentiation, subsequently restoring bone mass in DOP model.^23^, ^40^ Given the crucial role of Nlrp3‐mediated pyroptosis in the pathogenesis of DM and its associated complications, particularly the involvement of osteoblast pyroptosis in DOP progression,^23^, ^40^ therapeutic interventions targeting pyroptosis may offer a novel approach for the treatment of DOP. Encouragingly, both our team and other scholars have demonstrated the potent anti‐inflammatory effects of AP/PCC, which can inhibit Nlrp3‐mediated pyroptosis and ameliorate a variety of inflammatory disorders, including diabetic nephropathy and neuroinflammation, by reducing the levels of inflammatory factors, especially IL‐1β.^26^, ^27^, ^49^, ^50^, ^51^ Herein, our data showed that the aberrant activation of the Nlrp3‐mediated pyroptosis of osteoblasts within the vertebral bodies of STZ‐induced DM rats was accompanied by impaired osteogenic differentiation (evidenced by decreased levels of Runx2 and Osterix) and secretory capacity (evidenced by reduced Opg, Oc and Col1). Noteworthy, AR/PCC significantly inhibited osteoblast pyroptosis primarily by suppressing the aberrant upregulation of Nlrp3, Gsdmd and IL‐1β in the vertebral bodies of DM rats, thus facilitating osteogenesis and preventing bone loss in DOP progression. These findings substantiate the effective protective properties of AR/PCC against DOP through the inhibition of the Nlrp3‐mediated inflammatory process in the vertebral bodies of STZ‐induced DM rats.

Accumulating studies have elucidated the pivotal role of oxidative stress in the pathogenesis of DOP, and antioxidant therapy has been explored as a feasible therapy for DOP patients.^41^, ^52^, ^53^, ^54^, ^55^ The Nrf2/Keap1 pathway serves as a crucial regulator of the antioxidant response, orchestrating the expression of numerous antioxidant genes to maintain cellular redox homeostasis. It actively participates in various intracellular defence mechanisms, including the prevention of osteoblast apoptosis and the development of osteoporosis in individuals with DM.^52^, ^56^, ^57^ Emerging evidence from our own research along with others suggests that blockade of the Nrf2/Keap1 pathway induces pyroptosis of thyroid cells, and AR/PCC could effectively alleviate oxidative damage and reduce inflammation by re‐activating the Nrf2/Keap1 pathway.^33^, ^58^ Consistent with these findings, our study demonstrates AR/PCC treatment significantly counteracts the elevated expression of Keap1 and the reduced expression of Nrf2 in DM rats, indicating that AR/PCC can safeguard against oxidative stress in the vertebral bodies of DM rats. The above findings suggest that the inhibitory effect of AR/PCC on osteoblast pyroptosis is mediated through the re‐activation of the Nrf2/Keap1 pathway.

Besides, inflammation, angiogenesis and innervation are interconnected processes within osteoporosis that may synergistically affect disease progression. Kusumbe et al.^59^ have uncovered that the periphery of Cd31^+^ type H endothelium selectively harbours over 82% of Runx2^+^ and 70% of Osterix^+^ osteoprogenitors, and these endothelial cells could release osteogenic factors, such as BMP2, to promote osteoblast differentiation and mineralization.^60^ Conversely, mature osteoblasts produce Vegf, an angiogenic factor, to further support angiogenesis and improve bone structure by augmenting Alp and Oc levels.^61^ Previous studies have also revealed an increase in the number of Cgrp^+^ sensory nerves within active bone tissues, directly participating in bone remodelling via the modulation of local blood vessel blood flow,^62^, ^63^ highlighting the vital role of the peripheral nervous system in the regulation of osteogenic differentiation and osteogenesis. Consistent with the literature, we discovered bone mass loss and impaired osteogenic differentiation in the vertebral bodies of DM rats, accompanied by disrupted angiogenesis and nerve ingrowth. In addition, previous investigations utilizing the corneal neovascularization model and danazol‐induced precocious puberty model have demonstrated that AR/PCC could hinder Vegf‐mediated endothelial cell proliferation in HUVECs and suppress Netrin‐1 in hypothalamic tissues, indicating AR/PCC possesses the capability to inhibit angiogenesis and nerve ingrowth.^64^, ^65^ Unexpectedly, our study revealed AR/PCC treatment can restore or even reverse the reduction of angiogenesis and nerve ingrowth in DM rats. Therefore, the pharmacological effect of AR/PCC on angiogenesis and innervation remains contentious, and we speculated that this discrepancy may be attributed to the heterogeneous microenvironment of the vertebral body of the DOP model compared to the aforementioned two models, warranting further research.

Besides, there are several limitations in this study. In a previous study, by using a UPLCQTOF‐MS method, we have identified the potential active components of AR/PCC herb pair in serum, including mangiferin, tetrahydroepiberberine, phellodendrine, magnoflorine, 13‐hydroxyoxyberberine, palmatine and berberine.^11^ Herein, we have demonstrated the favourable anti‐osteoporosis effects of AR/PCC in the STZ‐induced rat DM model, which regulates imbalanced bone metabolic homeostasis by abrogating oxidative stress‐induced osteoblast pyroptosis. However, the precise interaction mechanisms between the active ingredients of AR/PCC and the Nrf2/Keap1 pathway, Nlrp3‐mediated pyroptosis of osteoblasts, as well as angiogenesis and nerve ingrowth, remain enigmatic and warrant further clarification. Moreover, the role of AR/PCC in mitigating impaired bone metabolism homeostasis under intrinsic conditions of oxidative stress, particularly through the reduction of FSG or other potential mechanisms, remains unclear. Therefore, deeper exploration is required to dissect the underlying reasons for impaired bone metabolic homeostasis in the presence of oxidative stress. Third, aside from the dysregulation of osteoblast‐related bone formation, the pathogenesis of osteoporosis in the context of DOP also involves additional factors such as osteoclast‐related bone resorption, disturbances in the mechanical environment, immune dysfunction and nutrition deficiency.^66^ However, the effects of AR/PCC regarding these aspects have yet to be unravelled, and future investigations are warranted to unravel the impact of AR/PCC on osteoclast activity, immune dysfunction, and nutrient availability.

## 5 CONCLUSION

In summary, our study illustrates the effectiveness of AR/PCC in mitigating the elevation of FSG, improving bone loss and osteogenic capacity, promoting nerve ingrowth and angiogenesis, and inhibiting the Nlrp3‐mediated pyroptosis in osteoblasts through the Nrf2/Keap1 pathway activation.

## AUTHOR CONTRIBUTIONS

**Fangda Fu:** Data curation (equal); formal analysis (equal); methodology (equal); writing – original draft (equal). **Huan Luo:** Data curation (equal); formal analysis (equal); methodology (equal); software (equal). **Yu Du:** Data curation (equal); formal analysis (equal); methodology (equal); software (equal). **Yuying Chen:** Formal analysis (equal); software (equal). **Kun Tian:** Formal analysis (equal); methodology (equal); software (equal). **Jin Pan:** Formal analysis (equal); methodology (equal); software (equal). **Jian Li:** Formal analysis (equal); methodology (equal); software (equal). **Nani Wang:** Formal analysis (equal); methodology (equal); software (equal). **Ronghua Bao:** Formal analysis (equal); methodology (equal); software (equal). **Hongting Jin:** Formal analysis (equal); methodology (equal); software (equal). **Peijian Tong:** Formal analysis (equal); methodology (equal); software (equal). **Hongfeng Ruan:** Conceptualization (lead); writing – original draft (equal); writing – review and editing (equal). **Chengliang Wu:** Formal analysis (equal); methodology (equal); software (equal); writing – original draft (equal); writing – review and editing (equal).

## FUNDING INFORMATION

This work was financially supported by National Natural Science Foundation of China (no. 82174140, 82174401, 81973870, 81973881), Natural Science Foundation of Zhejiang Province (no. LQ23H270003 and LY22H270003), Joint Funds of the Zhejiang Provincial Natural Science Foundation of China (no. LBY22H270008), Traditional Chinese Medical Administration of Zhejiang Province (no. 2023ZR019, 2023ZL128, 2022ZX005, 2022ZB119, 2021ZB090), Zhejiang Medical and Health Science and Technology Project (no. 2023RC194, 2021KY222), Research Project of Zhejiang Chinese Medical University (no. 2021JKZDZC02, 2021JKZKTS036A), Research Project of Zhejiang Chinese Medical University Affiliated Hospital (no. 2022FSYYZZ05 and 2022FSYYZQ02), Zhejiang Chinese Medical University School‐level Education and Teaching Reform Project (no. CY22001).

## CONFLICT OF INTEREST STATEMENT

The authors confirm that there are no conflicts of interest.

## Supporting information


Figure S1

## Data Availability

The original data supporting the conclusions of this article will be provided by the authors, without undue reservation.
