# Supplementary figures and images for: AR/PCC herb pair inhibits osteoblast pyroptosis to alleviate diabetes‐related osteoporosis by activating Nrf2/Keap1 pathway
Source: J Cell Mol Med. 2023 Aug 24;27(22):3601–13. doi: 10.1111/jcmm.17928 (PMC10660633; doi:10.1111/jcmm.17928)

Fig. S1

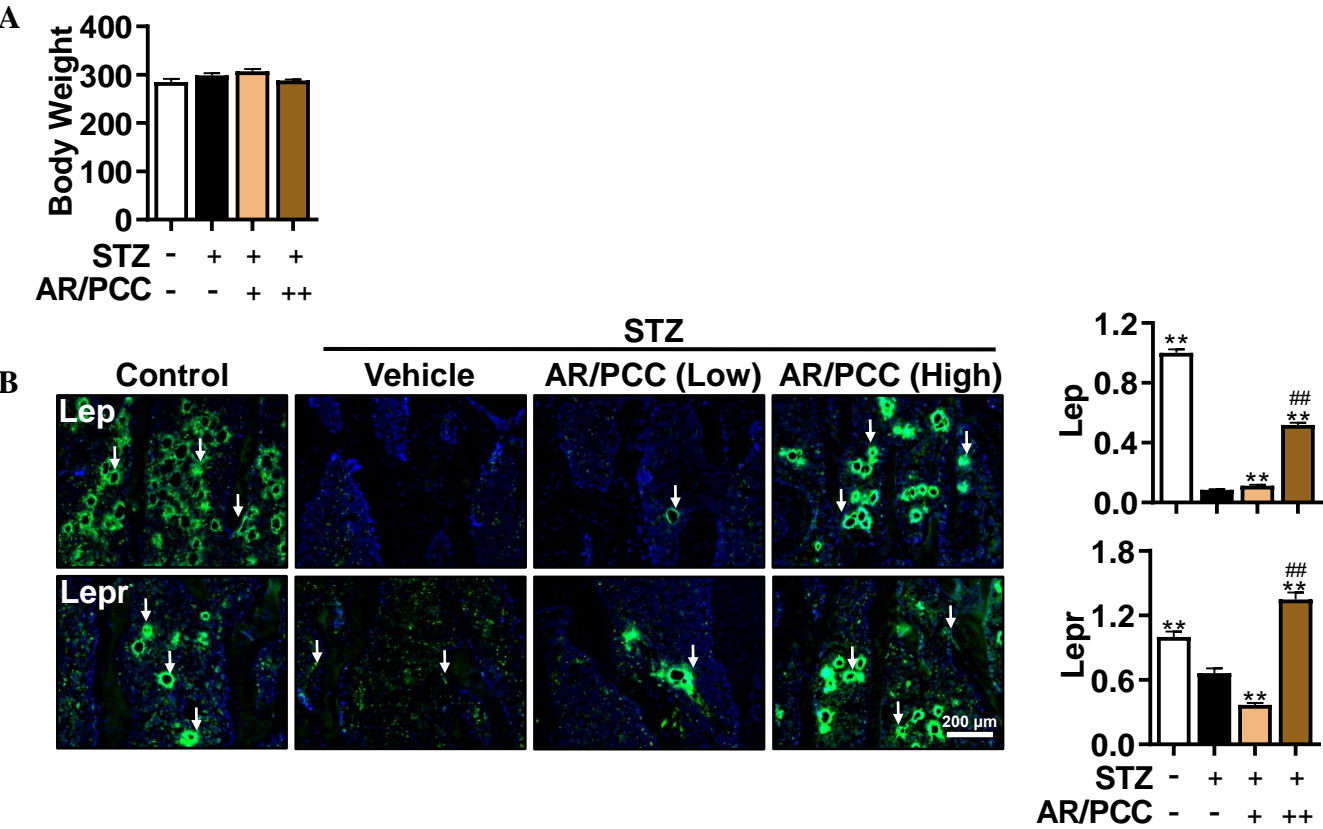

Supplement: Supplementary file 1 — Figure S1 [file JCMM-27-3601-s001.pdf]
